# Cognitive costs in motor-cognitive performance assessments depend on movement complexity and cognitive task design

**DOI:** 10.3389/fspor.2025.1482976

**Published:** 2025-06-05

**Authors:** Gülsa Erdogan, Lutz Vogt, Florian Giesche, David Friebe, Winfried Banzer, Andreas Mierau, Thorben Hülsdünker

**Affiliations:** ^1^ Department of Sports Medicine and Exercise Physiology, Goethe University Frankfurt, Frankfurt am Main, Germany; ^2^ Division of Preventive and Sports Medicine, Institute of Occupational, Social and Environmental Medicine, Goethe University Frankfurt, Frankfurt am Main, Germany; ^3^ Medical Department Eintracht Frankfurt Soccer AG, Frankfurt am Main, Germany; ^4^ Department of Sport, LUNEX, Differdange, Luxembourg; ^5^ Luxembourg Health & Sport Sciences Research Institute (LHSSRI), Differdange, Luxembourg

**Keywords:** executive function, motor-cognitive testing, reaction time, SKILLCOURT, motor-cognitive interference

## Abstract

**Objective:**

Cognitive performance is typically assessed using computer-based tests where participants respond via a simple upper extremity motor task such as a button press. This type of assessment has been criticised for its low ecological validity that does not consider the interaction between cognitive and more complex motor skills in sports and everyday life situations, which results in motor-cognitive interference. Consequently, motor-cognitive assessments integrating a more complex motor response into a cognitive test have gained popularity. However, the cognitive costs in motor-cognitive tests due to the interference of cognitive and motor processes have not yet been determined. Therefore, this study aimed to quantify the cognitive costs in motor-cognitive tests.

**Methods:**

Thirty-three healthy athletes performed four cognitive tests (simple reaction, choice-reaction, working memory, cognitive flexibility) in a cognitive and motor-cognitive setting. For the cognitive task, participants performed a computer-based cognitive assessment by responding with a button press on a keyboard. In the motor-cognitive condition, participants conducted a stepping movement which was identified by a Light Detection and Ranging (LiDAR) system integrated into motor-cognitive testing and training technology (SKILLCOURT®). Cognitive costs were determined by comparing reaction time and error rate between conditions (cognitive vs. motor-cognitive) while controlling for differences in measurement technology, neuromuscular conduction delay, and movement amplitude. Correlation analyses quantified the relationship between cognitive and motor-cognitive performance.

**Results:**

There were cognitive costs, as indicated by slower reaction times in the motor-cognitive test, for the choice-reaction (*p* = 0.014) and working memory (*p* < 0.001) tests. There were inverse cognitive costs, denoted by faster reactions, in the motor-cognitive compared with the cognitive condition for the cognitive flexibility test (*p* < 0.001). There were strong correlations for the simple-reaction (*r_31_* = 0.79, *p* < 0.001), choice-reaction (*r_31_* = 0.60, *p* < 0.001), and cognitive flexibility (*r_28_* = 0.83, *p* < 0.001) tests. The working memory task revealed a moderate correlation (*r_31_* = 0.46, *p* = 0.009).

**Conclusion:**

The results confirm the presence of cognitive costs in motor-cognitive assessments. The type of motor response and test design influence cognitive costs and test performance and can even result in inverse cognitive costs during motor-cognitive tasks. This must be considered when interpreting motor-cognitive tests and suggest that computer-based assessments cannot simply be replaced by motor-cognitive alternatives.

## Introduction

Cognitive functions play an important role in daily living and sports performance. A higher level of cognitive abilities such as decision-making, working memory, response inhibition, and cognitive flexibility contribute to academic success (1, 2), career success (3, 4), and better performance in sports (5, 6), as well as better quality of life and fall prevention in the elderly population (7, 8). In sports, athletes perform in cognitively demanding and dynamic environments. Athletes must react, make decisions, and adapt their motor behaviour in split seconds. The importance of cognitive performance in sports has been increasingly emphasised (9), and athletes have repeatedly been shown to outperform non-athletes in various cognitive abilities (10, 11). Moreover, cognitive performance as represented by executive functions is directly related to performance and success in football players (12, 13) as well as sport-specific skills in volleyball players (6). In addition to sport performance, cognition has a substantial impact on injuries. Based on previous research, it can be concluded that athletes with lower cognitive abilities experience a higher risk of injury (14, 15). Together, these findings support the integration of cognitive assessments into sport diagnostics to determine performance and injury risk (16, 17).

Due to the low ecological validity of widely used paper-pencil and computer-based cognitive assessments, the transfer of test results to real-life applications has repeatedly been questioned (18–20). Activities in daily life and sports are performed in dynamic and often unpredictable environments and involve complex motor actions such as locomotion, where neural resources must be shared between cognitive and motor tasks. This results in motor-cognitive interference (21–23) that manifests in decreased performance when combining cognitive and motor tasks compared with performing both tasks in isolation. Dual-task experiments on balance control and locomotion have repeatedly shown dual-task costs in motor-cognitive settings (21, 24, 25). Isolating cognitive abilities in paper-pencil or computer-based cognitive assessments does not account for this motor-cognitive interference.

The recent findings reported by Wilke et al. (26) support this critique. Although these authors tested the same cognitive ability, they found that performance correlates poorly when using an upper extremity (cognitive) or lower extremity (motor-cognitive) motor response. They suggested that cognitive and motor-cognitive assessments are largely independent of each other due to motor-cognitive interference effects in the more complex lower extremity response condition. Similar results have been reported in a sport-specific context. For example, it is well established that change of direction and reactive agility reflect different abilities and that correlations between agility and reactive agility are comparatively low (27). In the same vein, when researchers have attempted to transfer executive function tests on working memory, cognitive flexibility, and conflict inhibition from an upper extremity keyboard input to a football-specific motor response, they found rather low correlations indicating a maximum explained variance of only about 25% (28, 29). These findings suggest that computer-based cognitive assessments cannot simply be transferred to motor-cognitive tests. Instead, it appears that even in tasks with a low cognitive load (e.g., reactive agility) motor-cognitive interference occurs that manifest in cognitive costs.

There has been no study quantifying the cognitive costs in motor-cognitive assessments. Further, the abovementioned studies did not vary the motor response, the cognitive task (26), or the stimulus characteristics (28, 29). Thus, according to the concept of stimulus and task correspondence (30), it remains unclear to what extent the low correlations can be explained by the more complex motor action in motor-cognitive testing and what may be attributable to the stimulus and test setup. Moreover, motor-cognitive interference effects may depend on the task design: the greater the contribution of the motor part in a motor-cognitive task to performance, the lower the expected correlation between the cognitive and motor-cognitive condition. The same applies to the test design. Cognitive tests performed on a computer often use standardised or random interstimulus intervals between trials, while other apply adaptive protocols. It is well established that the response-stimulus interval (RSI, the time between the response and the presentation of the following stimulus) affects reaction time: a longer RSI, up to 500 ms, improves the reaction speed (31). The motor response of more complex motor tasks is more time consuming, so adaptive protocols especially may show lower cognitive costs because the anticipated interference between the cognitive and motor components may be balanced by advantages due to the longer RSI.

While there has been intense research in the field of dual-task paradigms and associated cognitive costs (32), only a few studies have addressed motor-cognitive tasks. This distinction is essential because dual-task assessment adds a cognitive demand to a motor task (e.g., walking while counting backwards) while both tasks remain independent. In this setting, the cognitive component can be considered a distractor (33) but not a prerequisite to complete the task (i.e., it is possible to walk without counting backwards and vice versa (34). In contrast, motor-cognitive tasks require successful completion of both the cognitive and motor parts (e.g., stepping left or right based on previous decision-making). By integrating the cognitive and motor components, motor-cognitive exercises are considered to achieve better ecological validity in both testing and training (29, 34). Given the increases popularity of motor-cognitive testing approaches in sport science and accumulating evidence that suggests existing cognitive tests should be replaced by alternatives integrating more complex and thus ecologically valid motor actions (28, 29), it is essential to identify potential cognitive costs that must be considered when interpreting the results. For practitioners, the magnitude of cognitive costs in motor-cognitive testing remains unclear based on previous correlation approaches. Further, it is necessary to determine how the relation between cognitive and motor-cognitive task performance depends on the relative contribution of the motor task to test performance and the influence of the test design (a fixed interstimulus interval vs. an adaptive protocol).

Although motor-cognitive interference is a well-established phenomenon, previous research has focused on dual tasks with low ecological validity. Further, studies aiming to estimate motor-cognitive interference in motor-cognitive assessments have been limited to correlation analyses lacking a direct estimation of the magnitude of cognitive costs. This study is first to quantify the cognitive costs of motor-cognitive testing by comparing a cognitive task performed in a computer-based setup (keyboard button press) to a motor-cognitive assessment that integrates a more complex stepping response into the cognitive test. In addition, the cognitive tasks varied between random, fixed, and adaptive interstimulus intervals to determine the effects of task design and RSI on cognitive costs. We hypothesised that the more complex lower extremity motor action in the motor-cognitive condition results in cognitive costs that manifest in a slower reaction time due to resource investment in postural and balance control. We expected that the correlations between both conditions increase with a higher relative contribution of cognition to test performance. Moreover, the short RSI for adaptive protocols, especially for the keyboard input, should at least partially balance the higher cognitive costs in the motor-cognitive task.

## Materials and methods

### Sample size calculation

An *a priori* sample size calculation [G*Power 3.1.9.7; (35)] was based on previously reported effect sizes (*η*_p_^2^) of 0.05 (36) and 0.54 (37) for postural stability and reaction tasks, respectively. Due to the heterogeneity of previous effects, a medium effect size (*η*_p_^2^ = 0.1) was selected for the calculation. Based on an alpha level of 0.05 and a test power of 0.8, this resulted in 16 participants for the one-way analysis of variance (ANOVA) and 21 participants for the one-sample *t*-test. However, for a reaction task, the relative contribution of the cognitive component is lower compared with more complex cognitive abilities. Because this study included reaction time as well as more demanding cognitive tasks such as cognitive flexibility, the differences between the cognitive and motor-cognitive condition were expected to be even smaller. Therefore, a sample that was large enough to determine differences between conditions even at a low effect size (*η*_p_^2^ = 0.06) was recruited (*n* = 27).

### Participants and ethics

Thirty-three healthy athletes from different sports (21 males, 22.7 ± 2.7 years, body mass index: 23.19 ± 2.45 kg/m^2^, years of experience: 9 ± 6 years, training load per week: 9.03 ± 5 h) volunteered to participate. The participants were recruited from the cohorts of sport and exercise science students at the university. Fifteen athletes participated in ball and team sports (football, tennis, basketball, etc.), 4 athletes performed endurance sports (swimming, running, and cycling), and 14 were assigned to other sports (cross-fit, horse riding, cheerleading, bouldern, fitness). All participants trained and participated in competitions regularly. Based on the classification system of McKay et al. (38), the athletes in this study are best defined as representing tier 2 (trained/developmental) and tier 3 (highly trained/national level). The exclusion criteria were limitations in daily activities, lower extremity injuries, and consumption of caffeine or alcohol on the day of testing. All participants were informed about the experimental protocol and their written consent was obtained. The Luxembourg National Research Ethics Committee (CNER) approved the study (202207/01 v2.0), which was performed in accordance with the Declaration of Helsinki (2013).

### Experimental protocol

**Figure 1:**
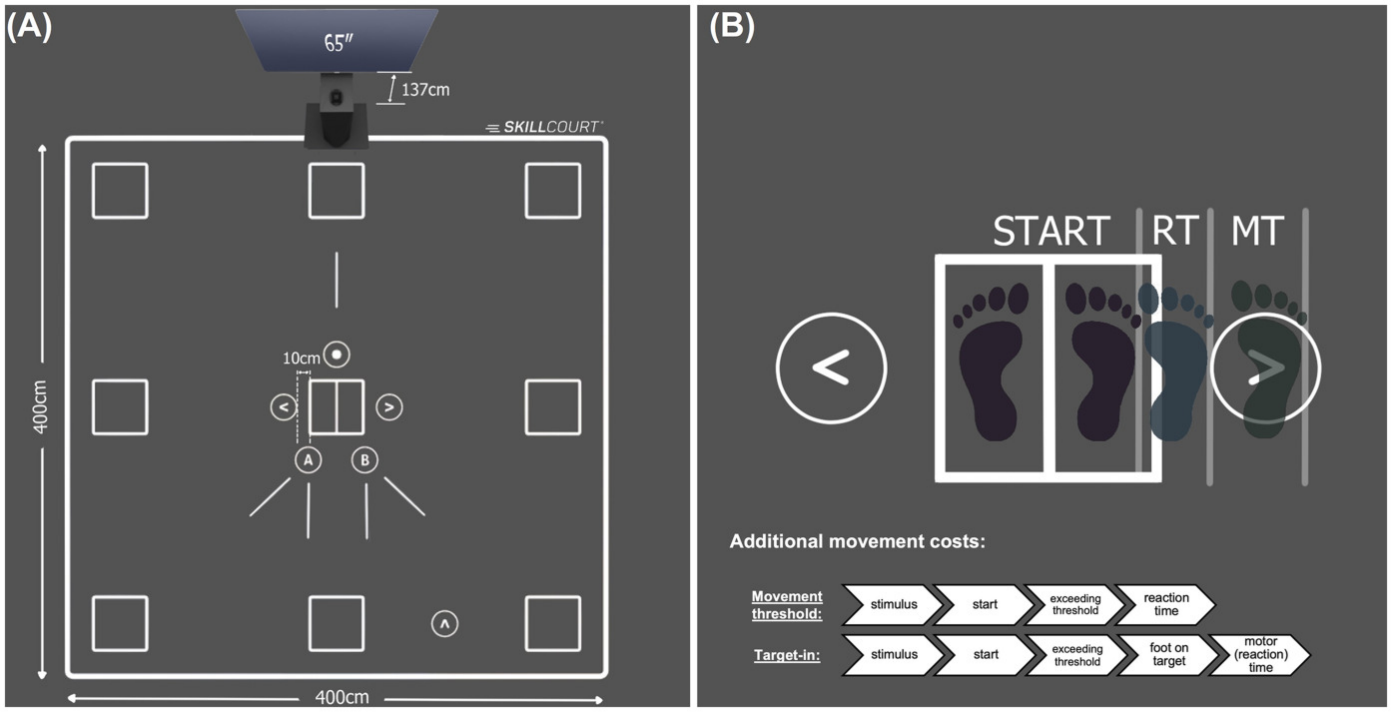
**(A)** setup of the skillcourt technology and dimensions of the court. **(B)** Illustration of determining reaction time and motor time on the Skillcourt. Reaction time is the time between stimulus presentation and exceeding the movement threshold while motor time represents the time between reaction time and reaching the target field (target-in).

**Figure 2:**
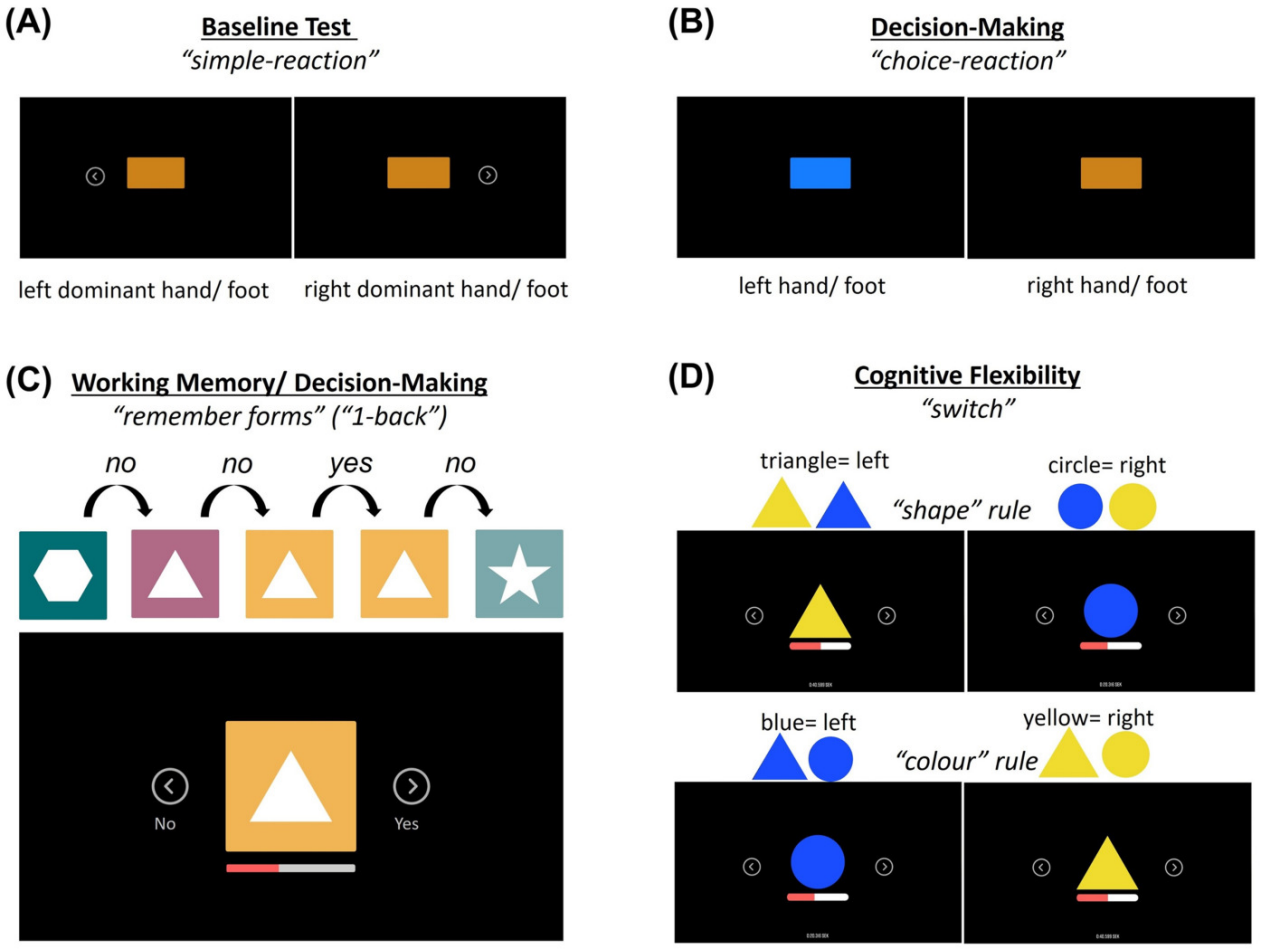
Illustration of the four tests conducted in the study: **(A)** the simple reaction test, **(B)** the two-choice decision-making test, **(C)** the 1-back test on working memory/decision making, and **(D)** the switch test on cognitive flexibility.

The participants visited the laboratory on two days. Both test sessions lasted for about 45 min and were conducted at least 24 h apart. To avoid the effects of circadian rhythm on test performance, the participants had to perform both tests at the same time of the day ± 3 h. The average time between the two test days was 2 days (±1.3 days). The average absolute difference between the test time on the two test days was 36 min (±48 min). All tests were conducted on the SKILLCOURT® (SKILLCOURT GmbH, Schweinfurt, Germany; Figure 1A), representing a valid (39) and reliable (40) technology for motor-cognitive testing and training. This technology uses a Light Detection and Ranging (LiDAR) system to continuously scan the participant's position on a 4 × 4 m court. A 50 m sub-maximal reactive agility task on the SKILLCOURT was conducted as a warm-up. Four tests (two per test day) were conducted to assess cognitive flexibility (switch test), working memory/decision-making (1-back test), and decision-making (choice-reaction test). A simple-reaction test, excluding cognitive contribution, served as a control condition (Figure 2).

This study applied a within-subjects design, where each participant completed all four cognitive tests that were administered on two test days (two tests per test day). Each test was performed in two response conditions, ’Skillcourt’ and ‘keyboard’. Except for the type of motor response (Skillcourt = lower extremity, keyboard = upper extremity), the tests were identical to ensure comparability of the cognitive demands. In the Skillcourt condition, the participants responded by activating the target fields left/ right of the centre field with the corresponding foot. In the keyboard condition, the participants made their inputs by pressing the left/ right arrow keys on the keyboard using the index finger of the corresponding hand. The keyboard condition required a minimal motor response by only pressing two arrow keys with the right and left index finger, which is comparable, or even less complex when compared with other computer-based assessments defined as ‘cognitive tests’ (41). Although it may be argued that due to the involvement of an upper extremity motor response, the task cannot be considered ‘cognitive’, it has been defined as a cognitive test due to its widely accepted use in cognitive training, also called ‘brain training’ (42). Accordingly, the keyboard input condition was considered to be the cognitive test condition. The term ‘motor-cognitive’ represents the integration of a more complex motor response into the cognitive task. Stepping movements on the SKILLCOURT require postural stability that in turn is associated with motor-cognitive interference (36); thus, it was considered to be the motor-cognitive condition.

For all tests, the participants stood in the centre field of the SKILLCOURT®. The keyboard was placed on a height-adjustable table in front of the participants. Before each test, the participants performed a test trial to familiarise themselves with the test. Two cognitive tests were conducted per day, with each response condition (keyboard and Skillcourt) conducted twice. To minimise order effects, the sequence of conditions (Skillcourt and keyboard) was alternated and counterbalanced and the sequence of tests (the simple-reaction, choice-reaction, 1-back, and switch tests) was counterbalanced across the participants. For each cognitive test and condition, the best result of the two assessment runs was included in the data analysis.

For the lower extremity condition, the inputs were either generated by a foot movement exceeding a pre-defined threshold (movement threshold), or when entering one of the target fields (target-in; see Figure 1B). The movement threshold was defined by the first foot movement that was detectable by the LiDAR scanner. Similarly, activation of the target field was defined as the moment the LiDAR recognised the foot entering the target field. Because the threshold method only works if the foot is stationary, it was applied to the simple-reaction and choice-reaction tests that used a random interstimulus interval of 3–5 s. This allowed identification of reaction time (stimulus presentation—movement threshold) and motor time (movement threshold—target activation). In contrast, the 1-back and switch tests were adaptive, and a new stimulus was presented once the participant returned with the foot to the centre field (Skillcourt condition) or released the keyboard button (keyboard condition). Therefore, using the movement threshold criterion was not possible. Instead, for the 1-back and switch tests, reaction time was defined as the time between stimulus presentation and activation of the target field. Differentiating between reaction and motor times in the simple-reaction test allowed the different approaches used to measure reaction time (threshold vs. target-in) to be controlled (see Equations 1–4).

To determine the cognitive costs in this experiment, the keyboard-based input serves as the cognitive task where performance is only determined by cognitive processes due to the very simple finger movement. The purely motor condition is the simple reaction test using the Skillcourt input where performance only depends on the speed of lower extremity movement without cognitive processing (one stimulus one response). Accordingly, reaction performance that exceeds the time predicted by the cognitive (keyboard input) and motor (Skillcourt input) time is considered to be attributable to motor-cognitive interference. The calculation of the cognitive costs following this scheme is described in Equations 1–4.

### Simple-reaction test

The participants were instructed to activate the target field (Skillcourt condition) or to press the arrow key (keyboard condition) as fast as possible when an orange rectangle appeared at the centre of the screen (Figure 2A). The simple-reaction test applied the ‘movement-threshold’ function and differentiated between reaction time (stimulus to movement threshold) and motor time (movement threshold to target-in). Thirty stimuli were presented, divided into two blocks of 15 stimuli. The interstimulus intervals were randomised between 3 and 5 s.

### Choice-Reaction test

In the choice-reaction test, a rectangle was presented in orange or blue at the screen centre (Figure 2B). Blue stimuli required activation of the left target field (Skillcourt condition) or pressing the left arrow key (keyboard condition), while an orange stimulus required a response to the right target field/arrow key. The test included 30 trials, subdivided into 2 × 15 trials, with a 15 s break in between. The chance of blue and orange objects was 50%/50%. The interstimulus interval was randomised between 3 and 5 s. The participants were instructed to make their inputs as fast as possible while avoiding errors.

### 1-back test (remember forms)

The 1-back test assessed the participants’ working memory and decision-making ability. A sequence of symbols differing in shape and colour was shown. The participants had to decide whether the displayed symbol matched (‘Yes’) or did not match (‘No’) in shape and colour the symbol shown one trial before (see Figure 2C) and to activate the corresponding target field (Skillcourt) or arrow key (keyboard). The 1-back test used the ‘target-in’ function, meaning that the reaction time was defined as the interval between stimulus presentation and entering the target field (see Figure 1B). The interstimulus interval was 500 ms. Each stimulus was displayed for a maximum duration of 3 s. The probability of ‘Yes’ and ‘No’ trials was 50%/50%. The test had a duration of 60 s. The participants were instructed to make inputs as fast as possible while avoiding errors.

### Switch test

The switch test assessed the participants’ cognitive flexibility. A triangle or a circle was displayed in either yellow or blue. For the ‘shape’ rule, the participants had to respond to the left target field/arrow key for triangles or to the right target field/arrow key for circles, independent of the symbol colour. The ‘colour’ rule required the participants to react to the left for blue objects and to the right for yellow objects, independent of the shape (Figure 2D). The rule changed every two objects. As for the 1-back test, the switch test used the ‘target-in’ input function. Two 60 s intervals were performed, with a 15 s pause in between. In contrast to the simple reaction and choice reaction tests, the switch test did not apply a fixed interstimulus interval; rather, it was adaptive. A new stimulus was shown after the button press (keyboard condition) or return to the centre field (Skillcourt condition). This setup was introduced to determine whether the task design (fixed interstimulus interval vs. adaptive test) has an influence on the cognitive costs. The participants were instructed to make their inputs as fast as possible while avoiding errors.

### Data analysis

To quantify the cognitive costs in the motor-cognitive condition, reaction time in the keyboard task was subtracted from the Skillcourt condition, which results in the additional time needed for the lower extremity input. However, the technology used to determine the reaction time (button press vs. the LiDAR system), longer signal transmission times to the lower extremity compared with the upper extremity, and differences in movement amplitude between the Skillcourt (see Figure 1B) and keyboard conditions must be considered as confounding variables.

To account for these factors, the simple-reaction task served as the control condition. This test purely depends on perceptual rather than cognitive processes. Any differences in reaction time between the upper extremity (keyboard) and the lower extremity (Skillcourt) input must be attributable to factors independent of cognition. Therefore, according to Equation 1, the simple-reaction time of the keyboard condition ($SRT_{KB}$) was subtracted from the Skillcourt condition ($SRT_{SC}$) to obtain the difference in the reaction time during the simple-reaction test ($SRT_{diff}$). The result reflects the additional time needed for Skillcourt inputs, which could be due to longer signal transmission and differences in reaction detection (button press vs. movement threshold). Accordingly, this time must be subtracted from the difference between the Skillcourt and keyboard conditions in the choice-reaction, 1-back, and switch tests. Spiegel et al. (43) reported that movement planning as well as movement execution are affected by cognitive load in an even more complex grasping task, so the individual motor time determined in the SRT was also considered for all cognitive conditions.

(1) $$SRT_{diff}=SRT_{SC}-SRT_{KB}$$

SRT_diff_ was calculated individually and subtracted from the difference between Skillcourt (*CR_SC_*) and keyboard (*CR_KB_*) in the choice-reaction (CRT) condition according to Equation 2. Any result >0 indicates additional cognitive costs in the choice-reaction condition that must be related to the longer cognitive processing time of the more complex motor task.

(2) $$Cognitive\;costs\;(CRT)=(CRT_{SC}-\;CRT_{KB})-SRT_{diff}$$

For the 1-back and switch tests in the Skillcourt condition, the reaction time was determined based on the target-in input (Figure 1B). Therefore, in addition to SRT_diff_, the motor time (MT), representing the additional time needed in the Skillcourt condition for movement execution beyond the button press in the keyboard condition, must be considered. The cognitive costs for the 1-back and switch tests were calculated according to Equations 3 and 4, respectively.

(3) $$Cognitive\;costs\;(1back)=(1back_{SC}-\;1back_{KB})-(SRT_{diff}+\;MT)$$

(4) $$Cognitive\;costs\;(switch)=(switch_{SC}-\;switch_{KB})-(SRT_{diff}+\;MT)$$

The results reflect the difference in reaction time between the Skillcourt and keyboard input conditions while controlling for technological (reaction trigger), physiological (signal transmission), and motor (movement amplitude) components. Accordingly, the remaining discrepancies in the reaction must be attributable to cognitive processing. Any participants with cognitive costs exceeding ±2 standard deviations of the group average were excluded from the analysis. In addition to the reaction time, error rates (in %) were calculated for the Skillcourt and keyboard conditions and all cognitive tests.

### Statistical analysis

Statistical analyses were performed in JASP (55). The Shapiro–Wilk test confirmed that all variables followed a normal distribution. To identify cognitive costs in the Skillcourt condition, one-sample t-tests against 0 were used for all cognitive assessments. Because the keyboard condition served as the baseline, any significant difference from 0 would indicate positive (>0) or inverse (<0) cognitive costs in the Skillcourt condition. An additional repeated-measures ANOVA with the within-subject factor test (1-back, switch, and choice reaction) identified differences in cognitive costs across tests. A two-factor repeated-measures ANOVA with the factors condition (Skillcourt and keyboard) and test (1-back and switch) investigated differences in the error rates. Mauchly’s test assessed sphericity; the Greenhouse–Geisser correction was applied if sphericity was violated. The Bonferroni correction was applied to pairwise *post hoc* testing. To assess a direct relation between performance in the Skillcourt and keyboard condition, as reflected by the reaction time, Pearson correlation coefficients were calculated for all tests. The effect size was considered small (*d* > 0.2, *η_p_^2^* > 0.01, *r* > 0.1), medium (*d* > 0.5, *η_p_^2^* > 0.06, *r* > 0.3), or large (*d* > 0.8, *η_p_^2^* > 0.14, *r* > 0.5). The significance threshold was set to *p* < 0.05.

## Results

### Cognitive costs

**Figure 3:**
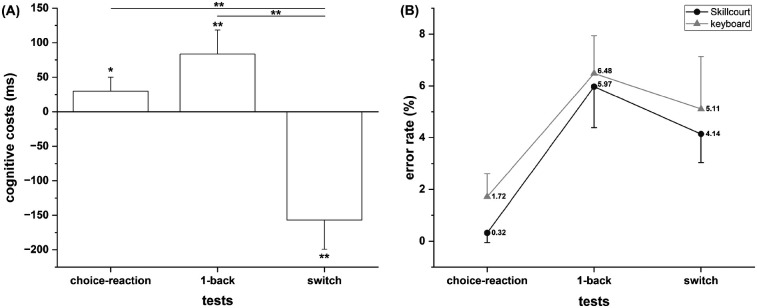
**(A)** cognitive costs illustrated as the difference between the cognitive (keyboard input) and motor-cognitive (skillcourt input) conditions for the three cognitive tests. **(B)** Comparison of the error rates between the conditions (keyboard vs. Skillcourt) and across cognitive tests. *p < 0.05; **p < 0.01.

Cognitive costs are defined as higher reaction times in the motor-cognitive condition compared with the keyboard condition after correcting for technological (reaction trigger), physiological (conduction delay), and motor (movement amplitude) differences. Inverse cognitive costs reflect faster reactions in the motor-cognitive condition. There were cognitive costs in the choice-reaction test [*t*_(30)_ = 3.74, *p* = 0.014, *d* = 0.51] and 1-back test [*t*_(29)_ = 4.95, *p* < 0.001, *d* = 0.9], as indicated by a slower reaction for the Skillcourt (motor-cognitive) condition compared with the keyboard input (cognitive) condition. However, there were inverse cognitive costs for the switch test, as indicated by faster reactions for the Skillcourt condition compared with the keyboard condition [*t*_(28)_ = –7.62, *p* < 0.001, *d* = −1.42]. ANOVA revealed significant differences in cognitive costs across tests [*F*_(2,50)_ = 64.85, *p* < 0.001, *η_p_^2^* = 0.72], with the 1-back (*p* < 0.001) and choice-reaction (*p* < 0.001) tests showing significantly higher cognitive costs compared with the switch test (Figure 3A).

### Error rate

There were higher error rates for the 1-back test compared with the switch test [*F*_(1,26)_ = 6.84, *p* = 0.015, *η_p_^2^* = 0.21], whereas the factor condition [*F*_(1,26)_ = 1.03, *p* = 0.32, *η_p_^2^* = 0.038] and the condition × test interaction [*F*_(1,26)_ = 0.07, *p* = 0.79, *η_p_^2^* = 0.003] remained insignificant, indicating no difference in the error rate between the keyboard and Skillcourt conditions (see Figure 3B).

### Correlation analyses

**Figure 4:**
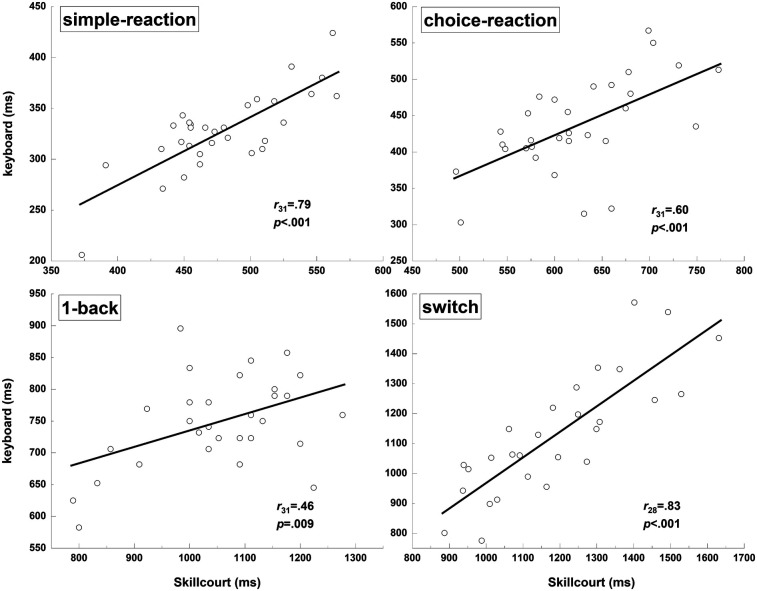
Correlation analysis of reaction times obtained for the keyboard and skillcourt conditions across all tests.

The correlations were strong for the simple-reaction (*r_31_* = 0.79, *p* < 0.001), choice-reaction (*r_31_* = 0.60, *p* < 0.001), and switch (*r_28_* = 0.83, *p* < 0.001) tests, while there was only a moderate correlation for the 1-back test (*r_31_* = 0.46, *p* = 0.009) (Figure 4).

## Discussion

This study is the first to quantify cognitive costs in motor-cognitive compared with cognitive assessments. The comparison controlled for confounding factors such as the testing technology, differences in signal transmission delay to upper and lower extremity, as well as the motor response amplitude. There were cognitive costs in the motor-cognitive condition in the choice-reaction and 1-back tests. While this finding is in line with the motor-cognitive interference model, the inverse cognitive costs in the switch test indicate that task design influences cognitive costs in motor-cognitive testing. These results suggest that cognitive tasks combined with a more complex motor response (motor-cognitive task) induce cognitive costs that depend on the task design. The motor-cognitive interference effect increases especially with a higher contribution of the motor task to the overall test performance. Further, cognitive costs depend on the task design and are higher in tests with fixed interstimulus intervals compared with adaptive tests. This information is essential for practitioners aiming to apply motor-cognitive testing and suggests that computer-based cognitive tests cannot simply be replaced by motor-cognitive assessments.

As hypothesised, there were cognitive costs for the more complex lower extremity motor tasks in the motor-cognitive condition compared with the keyboard condition. This was reflected in slower reaction times, a finding that supports the theory of cognitive-motor interference (21), which states that cognitive resources are shared between the cognitive and motor tasks. In fact, researchers have repeatedly shown that cognitive resources are required for postural and balance control, which interferes with cognitive performance (24, 25, 36). Furthermore, motor-cognitive interference increases with more complex movements. Reiser et al. (44) reported an increase in response time in an oddball reaction paradigm when changing from a standing to a walking task. They attributed this increase to a lower availability of resources for cognitive performance with a higher motor load. These findings and interpretations are in line with the present study. The shift from a double-support to a single-support stance in the motor-cognitive (Skillcourt) condition substantially increased the motor control demands (i.e., balance and posture control) compared with a continuous double-support stance in the cognitive (keyboard) condition. Therefore, it appears plausible to assume that the cognitive costs in the motor-cognitive assessments (the choice-reaction and 1-back tests) can be explained by the additional resource investment in motor control and lower resource availability for cognitive processing. However, future research will have to elaborate on the neural basis of the observed prolonged reaction times.

Interestingly, there were inverse cognitive costs for the motor-cognitive assessment in the cognitive flexibility (switch) test. This may be surprising given that postural and balance demands were similar to the choice-reaction and 1-back tests, which should result in comparable cognitive-motor interference. However, the results appear plausible when considering the task design and especially the RSI, which defines the time between the response and the occurrence of the next stimulus (45). A longer RSI [up to about 500 ms (31);] reduces reaction time, as has been shown in Stroop (46), choice reaction (45), and task-switching (47) paradigms; the latter is similar to the switch test used in this study. Further, the participants reacted more slowly and made more errors at a shorter RSI. In this study, the next stimulus was presented when the arrow key was released (keyboard) or when the participant returned to the centre field (Skillcourt). For the Skillcourt condition, the participants had to first move their foot back from the target field to the centre field, resulting in a substantially longer RSI compared with the keyboard condition. According to the refractory period (48) and advance configuration (31, 49), it can be assumed that advance configuration was not finished in the cognitive condition due to the very short RSI, which in turn delayed the response time for the following stimulus. These findings highlight that in addition to the complexity of the motor task, the cognitive test design can have a substantial influence on performance. Importantly, differences in the RSI only apply to the switch test. In the choice-reaction task, the RSI varied randomly from 2 to 5 s, while in the 1-back test, it was fixed at 500 ms. In both tasks, a random or fixed interstimulus interval with a minimum duration of 500 ms was used to provide sufficient time for advance configuration in both the cognitive (keyboard) and motor-cognitive (Skillcourt) conditions. In contrast, the adaptive protocol applied in the switch test resulted in a shorter RSI during the cognitive (keyboard) condition compared with the motor-cognitive (Skillcourt) condition. As a result, the associated delay in reaction time due to incomplete advance configuration in the cognitive task outweighs the cognitive costs of the motor-cognitive condition, resulting in the observed inverse cognitive costs for the switch test.

There was no difference in the error rates between the cognitive and motor-cognitive tests, suggesting that cognitive costs in the choice-reaction and 1-back tests manifest as longer reaction times and not increased error scores. Because error rates typically increase with very short interstimulus intervals and RSIs (50, 51), the participants likely invested more time in the keyboard condition of the switch test to ensure correct decision-making. This further supports the abovementioned hypothesis of incomplete advance configuration, which increased the reaction time and contributed to the inverse cognitive costs. This interpretation is consistent with the concept of speed-accuracy trade-off in cognitive tasks (52).

As expected, the correlations were relatively low for the choice-reaction (*r* = 0.6) and 1-back (*r* = 0.46) tests compared with the switch test (*r* = 0.83, *p* < 0.036). This can be explained by differences in the relative contribution of the cognitive and motor parts to the test performance in the motor-cognitive condition. While the motor component across motor-cognitive tests stays constant (stepping movement), the cognitive contribution is substantially higher in the switch test, as manifested in longer reaction times compared with the choice reaction (+650 ms) and 1-back (+250 ms) tests. Accordingly, the switch test, with a higher relative cognitive contribution, correlated more strongly to the cognitive condition (keyboard input). Overall, the correlation coefficients from the present study are higher than in the study by Wilke et al. (26) and more in line with a previous study (17) in which the authors used identical simple- and choice-reaction tasks (*r* = 0.69–0.76). The findings indicate that the relative contribution of the motor response to task performance has a major impact on the relation between cognitive and motor-cognitive tasks. The stronger the contribution of the motor component to the overall task performance (reaction time), the lower the observed correlation. In this context, the strong correlation for the simple-reaction test (*r* = 0.79) may be surprising because the contribution of the motor component should be highest in this condition. However, as the participants knew the motor task in advance (one stimulus and one response), they had already completed their posture and balance adjustments before they executed their movements. Accordingly, the impact of the motor task on the correlation was lower.

## Limitations

While this study provides valuable insights into motor-cognitive diagnostics and associated cognitive costs, several limitations should be acknowledged to contextualise the findings and to guide future research. First, as this study included athletes from various sports, which increases the generalisability of the findings to athletes in general, the results may not fully apply to sport-specific populations. Future research should address this limitation by investigating sport-specific populations and examining the effects of motor-cognitive assessments on parameters relevant to sport performance. This may also include higher stimulus and task correspondence for the motor-cognitive task to increase sport specificity. From a methodological perspective, using electromyography (EMG) could provide a more precise estimate of reaction time differences between cognitive and motor-cognitive tasks. As the movement amplitude was larger for the stepping task on the Skillcourt, there may have been variability between participants in movement execution, which may have affected especially the correlation between the cognitive and motor-cognitive conditions. Moreover, while the behavioural results support motor-cognitive interference and cognitive costs in motor-cognitive tasks, electroencephalography (EEG) may provide further insights into the neural basis of this phenomenon. EEG was not included in this study due to the higher number of required stimuli to obtain a sufficient signal-to-noise ratio. Future research should consider electrophysiological measures (EMG and EEG) to support the precision of the cognitive cost estimation and to unravel its neural mechanisms. Finally, while the study focused on athletes, the findings may also be relevant to the elderly population. Based on the literature, the elderly perform worse in dual-task paradigms compared with younger adults (53, 54). Based on the results, there should be further investigation to assess cognitive costs in motor-cognitive tasks.

### Implications for practice

The cognitive costs observed in this study indicate that computer-based cognitive assessments cannot simply be replaced by motor-cognitive tasks, as has been argued previously (28, 29). Instead, the cognitive costs depend on the relative contribution of the motor component to the test performance and may even be inverse if using an adaptive test design rather than a fixed interstimulus interval. While motor-cognitive tests have been proposed to offer higher ecological validity compared with computer-based assessments, their validity related to cognitive performance testing is often limited due to the inclusion of more complex motor actions. For example, as shown by Knöbel and Lautenbach (28), performing a working memory task integrating a soccer-specific motor response resulted in low explained variance (approximately 20%). Similar results have been reported for inhibition and cognitive flexibility tasks performed in a soccer-specific setting (29). Athletes and practitioners should be aware that cognitive and motor-cognitive tests assess different constructs that are influenced by motor-cognitive interference depending on the tested cognitive ability, task design, and relative contribution of the motor component to test performance. Such factors should be considered when interpreting motor-cognitive assessments and designing training programmes. This does not argue against the value of motor-cognitive assessments and the suggestion of their higher ecological validity. However, the transferability of motor-cognitive test results to more realistic indicators of sport performance (e.g., game metrics) compared with computer-based cognitive tests needs to be investigated in future studies.

## Conclusion

This study indicates that adding a more complex motor response (stepping movement) to cognitive tasks (i.e., motor-cognitive task) results in cognitive costs that manifest as an increased reaction time. Importantly, the type of motor response as well as the test setup, as reflected by the interstimulus interval and the relative contribution of cognitive and motor components to task performance, affect the magnitude of cognitive costs. These factors need to be considered when designing and interpreting motor-cognitive tests and emphasise that computer-based cognitive tasks cannot simply be replaced by motor-cognitive assessments.

## Data Availability

The raw data supporting the conclusions of this article will be made available by the authors, without undue reservation.
